# St. Gallen International Breast Cancer Consensus-Based Clinical Decision Validation: Concordance Assessment Between Deep Large Language Model Outputs and Global Expert Panel Recommendations

**DOI:** 10.1245/s10434-026-19176-1

**Published:** 2026-02-10

**Authors:** Yi Pan, Chenglong Duan, Jinsui Du, Jianing Zhang, Keyuan Du, Chenrong Zhang, Zhihao Liu, Wei Zhang, Bin Wang, Yu Ren, Zhao Sun, Lizhe Zhu

**Affiliations:** 1 Department of Breast Surgery, The First Affiliated Hospital of Xi’an Jiaotong University, Xi’an, Shaanxi Province China; 2 School of Cyber Science and Engineering, Zhengzhou University, Zhengzhou, Henan Province China; 3 Systems Engineering Institute, Xi’an Jiaotong University, Xi’an, Shaanxi Province China

**Keywords:** DeepSeek, St. Gallen international breast cancer conference, Breast cancer, Large language models, Clinical decision-making

## Abstract

**Background:**

The newly developed large language model (LLM) DeepSeek has shown potential for application in other medical fields. However, few systematic studies have assessed its concordance with international expert consensus or compared its performance with leading models such as Gemini 2.0 Pro and ChatGPT-4o in breast cancer.

**Materials and Methods:**

A total of 139 consensus questions from the 19th St. Gallen International Breast Cancer Conference (SG-BCC) were included into analysis. Each model was trained to answer each consensus question five times. The DeepSeek model was compared with the expert panel consensus in terms of concordance rate, robustness of the answers, Pearson correlation coefficient *r* for non-binary questions, and absolute proportion difference for binary questions. At the same time, a horizontal comparison was made with the previous LLMs Gemini 2.0 Pro and ChatGPT-4o.

**Results:**

The overall concordance rate between DeepSeek-V3 and the expert panel consensus was 63.31%, and the average answer robustness (i.e., its self-consistency across repeated queries) of DeepSeek-V3 was 86.69%. In addition, DeepSeek-V3 performed similarly to Gemini 2.0 Pro and ChatGPT-4o in terms of concordance rate of the most frequent answers (*p* = 0.849). In terms of model robustness, there were significant statistical differences among the models (*p* < 0.001), with DeepSeek-V3 significantly outperforming Gemini 2.0 Pro (*p* = 0.005) and ChatGPT-4o (*p* < 0.001).

**Conclusions:**

DeepSeek models showed moderate concordance in following the consensus of breast cancer expert panel and showed significant advantages in answer robustness, suggesting that DeepSeek has great application potential in the field of clinical decision-making for breast cancer.

**Supplementary Information:**

The online version contains supplementary material available at 10.1245/s10434-026-19176-1.

Breast cancer is one of the most common malignancies in women worldwide.^1–3^ This heterogeneous disease contains multiple subtypes and has complex and diverse treatment methods, posing a major threat to women’s health.^4–6^ The accelerated development of many effective new therapies has significantly improved the survival rates of all breast cancer subtypes.^7–12^ To optimize individualized treatment strategies, it is essential to continuously monitor and integrate the latest research results. Several international organizations, including the American Society of Clinical Oncology (ASCO), the European Society for Medical Oncology (ESMO), the National Comprehensive Cancer Network (NCCN), and the St. Gallen International Breast Cancer Conference (SG-BCC) expert panel, regularly publish evidence-based clinical practice guidelines.^13–16^ These organizations rigorously evaluate and discuss important international clinical studies to develop consensus recommendations for breast cancer diagnosis and treatment, thereby providing expert guidance for personalized treatment methods.

The biennial St. Gallen International Breast Cancer Conference convenes a global panel of breast cancer experts who conduct anonymous real-time voting on controversial or evidence-limited aspects of early breast cancer management. The final consensus reflects the collective opinions of top international experts, simulates the complex clinical decision-making processes, and provides valuable guidance for clinical practice.^16^

However, it remains a challenge for surgical oncologists to apply evolving and detailed consensus recommendations to individual patients. The increasing volume and complexity of clinical data require considerable time for review. This is especially evident when clinicians prepare for multidisciplinary tumor board discussions or manage uncommon clinical presentations. At the same time, artificial intelligence technologies represented by large language models (LLMs) have made breakthrough progress in recent years, and have shown great application potential in the medical field.^17–22^ They can rapidly integrate guideline-based treatment options for complex cases and support the preparation of multidisciplinary tumor board discussions.^23^ In regions with limited medical resources, LLMs may provide standardized and accessible clinical reference information. They can also serve as educational tools for trainees and assist in developing patient-oriented instructional materials.^24^ However, their clinical value depends on a fundamental requirement: they must be able to reproduce expert consensus in a consistent and accurate way. Therefore, careful evaluation of their performance against established clinical standards is essential before they can be considered for routine use. Among them, the newly developed and cost-effective large language model DeepSeek has also performed well in other medical fields.^25–28^ However, few systematic studies have assessed its concordance with international expert consensus in breast cancer clinical decision-making. According to the developer’s documentation, the model is available in two versions: DeepSeek-V3 and DeepSeek-R1. Each version has a different performance focus, and their usefulness in breast cancer clinical decision-making remains unclear. Therefore, this study evaluates both versions and compares the better-performing one with leading models such as Gemini 2.0 Pro and ChatGPT-4o.

## Materials and Methods

### Data Source

The question set for this study was derived from the consensus voting results presented at the 19th SG-BCC, which were obtained by one of the authors in this study who attended the conference. After excluding questions about previous clinical practice choices that could not be answered by LLMs, 139 consensus questions with clear answer options were finally included in the study. At the same time, an expert panel cohort was established on the basis of the expert panel voting results.

### Large Language Model Testing

We selected representative LLMs released before 15 March 2024 to evaluate their clinical decision-making performance. The models included DeepSeek, ChatGPT-4o, and Gemini 2.0 Pro. DeepSeek provides two versions: DeepSeek-V3, a general-purpose model, and DeepSeek-R1, which is optimized for complex reasoning. These four models have distinct features within the fast-evolving field of medical artificial intelligence (AI). Detailed information on their technical specifications and previous medical evaluations is provided in Supplementary Text 1.

All LLMs were tested through their official application programming interfaces (APIs) or publicly available online platforms. Before testing, we standardized the training process by requiring each model to use only knowledge available before 15 March 2025 (i.e., before the 19th SG-BCC). We provided each model with the NCCN Clinical Practice Guidelines: Breast Cancer (3rd edition, 2025)^15^ and the Chinese Society of Clinical Oncology (CSCO) Breast Cancer Diagnosis and Treatment Guidelines 2024^29^ for reference when answering questions.

To minimize bias from question phrasing, we reformatted each consensus question into standardized instructions before entering it to the LLMs. The prompts were as follows: “Please answer the following questions based on the above two guidelines, using data before March 15, 2025, and clearly provide the letter corresponding to the option you selected.” During testing, each model was independently run five times per question to establish a cohort of LLMs.^30^ The answer that appeared most frequently across these runs was defined as the “most frequent answer.” If the most frequent answer did not emerge in the first five tests, we conducted a sixth test. Each test started a new round of dialogue to eliminate memory interference caused by the previous question.

### Metrics

Concordance rate: The proportion of questions where the model’s most frequent answer agrees with the option with the highest proportion of expert panel votes.

Robustness: The proportion of the model’s most frequent answer that was selected in five (or six) conversations for a specific question. This metric can provide important supplementary information. It reflects the reliability and stability of the model’s outputs, which is essential for any tool intended for clinical use. A high robustness score indicates that the model produces consistent answers and that its outputs are not driven by randomness. It also suggests stronger internal confidence in the conclusions generated. In addition, examining robustness across different clinical topics helps identify areas where the model is stable or uncertain. Such findings are useful for guiding the development of safer and more effective AI systems for clinical decision support.

Absolute proportional difference: Specifically used for binary problems. This metric uses the option with the highest proportion of expert votes as the reference and calculates the absolute difference between the proportion of the option selected by the model and the proportion of experts voting for that option. The smaller the value, the closer the proportion predicted by the model is to the expert consensus.

Pearson correlation coefficient *r*: Specifically used for nonbinary problems. It measures the linear correlation between the distribution vector of the model’s answers and the distribution vector of the proportion of expert votes, quantifying the strength of the association between them.

### Statistical Analysis

In this study, since most measurement data (including robustness, absolute proportional difference, and Pearson correlation coefficient *r*) were non-normally distributed, they were expressed as medians and interquartile ranges (IQRs), and nonparametric tests were employed. Only in Table 2, to present detailed differences, were some indicators reported as means without accompanying statistical inference. Categorical data were expressed as frequencies (*n*) and percentages (%). The Pearson chi-squared test was used to compare concordance rates between models. For nonparametric analyses, the Kruskal–Wallis test was applied. When Kruskal–Wallis results were statistically significant (*p* < 0.05), post hoc comparisons were performed using the Dwass–Steel–Critchlow–Fligner (DSCF) method. All statistical tests were two-sided, with a significance level of *α* = 0.05. Analyses were conducted using SPSS PASW Statistics 18, R software (version 4.2.2), and MSTATA software (https://www.mstata.com/).

### Ethical Approval

This study does not involve any ethnic human participant data. According to national laws and regulations and institutional requirements, this study does not need to be reviewed and approved by the ethics committee.

## Results

### Characteristics of the Research Question Set

A total of 139 questions were included in this study, covering various areas of breast cancer diagnosis and treatment. According to the number of options for each question, the questions were divided into binary questions (*n* = 37, 26.62%) and nonbinary questions (*n* = 102, 73.38%). In addition, from a clinical perspective, the questions were divided into nine main topics (Table 1).

**Table 1:** Distribution of research question characteristics

Category	Subcategory	Number of cases (*N*)	Percentage (%)
Total		139	100.00
*Problem Type*			
	Binary questions	37	26.62
	Nonbinary questions	102	73.38
*Topic*			
	Ductal carcinoma in situ	10	7.19
	Genetic testing	17	12.23
	Breast and axillary surgery	16	11.51
	Radiation therapy	28	20.14
	Systemic therapy: ER positive, HER2 negative breast cancers	44	31.65
	Systemic therapy: HER2 positive breast cancer	10	7.19
	Oligometastatic breast cancer	7	5.04
	Treatment of local-regional recurrence of breast cancer	2	1.44
	Survivorship	5	3.60

Binary questions are questions with two options and nonbinary questions are those with three or more options

### Comparison of DeepSeek Model with Expert Panel

The first stage of the analysis compared DeepSeek-V3 and DeepSeek-R1 to determine which model was more suitable for further evaluation. Across the 139 SG-BCC questions, DeepSeek-V3 showed a higher overall concordance rate with the expert consensus and greater robustness than DeepSeek-R1. It also performed better in key stratified analyses. On the basis of these findings, DeepSeek-V3 was selected as the primary model for all subsequent analyses in the main text. Detailed results for DeepSeek-R1 are provided in Supplementary Table 1.

Among the 139 questions, DeepSeek-V3’s answers were consistent with the options with the highest proportion of expert panel votes in 88 questions (63.31%). For these 88 questions, the model showed an average robustness of 90.23%, which means that in five (or six) rounds of answering, DeepSeek-V3 selected the most frequent expert vote in 90.23% of cases. Across all 139 questions, the answers provided by DeepSeek-V3 had an average robustness of 86.69%. Out of a total of 139 questions, DeepSeek-V3 answered 109 questions (78.42%) with a robustness of at least 80%. Among the 88 consistent questions, expert panel voted with an average majority of 65.62%. Among all the questions, 25 questions had a voting rate of more than 80% (Table 2).

**Table 2:** Detailed comparison of DeepSeek-V3 with the 19th St. Gallen International Breast Cancer Conference Expert Panel

	DeepSeek-V3	Expert panel
*Overall performance (N = 139)*		
Common responses	88 (63.31%)	
Average robustness/majority in all questions (*N* = 139)	86.69%	62.01%
Average robustness/majority in common answers (*N* = 88)	90.23%	65.62%
Questions answered with average robustness/majority of ≥ 80%	109 (78.42%)	25 (17.99%)
Performance by question type		
*Binary questions (N = 37)*		
Common responses	25 (67.57%)	
Absolute proportional difference^1^	0.30 (0.13, 0.50)	
*Nonbinary questions (N = 102)*		
Common responses	63 (61.76%)	
Pearson correlation coefficient *r*^1^	0.82 (0.20, 0.98)	
*Comparison of robustness among LLMs by topic classification*		
Topic “Systemic therapy: ER positive, HER2 negative breast cancers” (*N* = 44)		
Common responses	22 (50.00%)	
Average robustness/majority in this topic	84.09%	58.34%
*Topic “Genetic testing” (N = 17)*		
Common responses	13 (76.47%)	
Average robustness/majority in this topic	87.06%	67.71%
*Topic “Radiation therapy” (N = 28)*		
Common responses	15 (53.57%)	
Average robustness/majority in this topic	85.00%	64.04%

^1^Median (M) and interquartile range (P25, P75) of the above indicators

In this table, most of the measurement data are not normally distributed, but robustness is reported in the form of mean to reflect the specific differences without statistical inference. Absolute proportional difference and Pearson correlation coefficient *r* are still expressed in median and interquartile range

*LLMs* large language models

In addition, this study conducted a stratified analysis of different question types and specific clinical hot topics, revealing subtle differences in the performance of DeepSeek-V3. Among the 37 binary questions, 25 answers of DeepSeek-V3 were consistent with the options with the highest proportion of expert panel votes, accounting for about 67.57%. The median absolute proportion difference of the answers was 0.30. Among the 102 nonbinary questions, 63 answers of DeepSeek-V3 (accounting for 61.76%) were consistent with the options with the highest proportion of expert panel votes, with a median Pearson correlation coefficient *r* of 0.82. In addition, in the three clinical hot topics with the largest number of questions, “Systemic therapy: ER positive, HER2 negative breast cancers,” “Genetic testing,” and “Radiation therapy,” the concordance rates of DeepSeek-V3 answers were 50.00%, 76.47%, and 53.57%, respectively (Table 2).

The results suggest that the DeepSeek-V3 model exhibits moderate concordance in following the opinions of the SG-BCC expert panel and has high robustness in its responses.

### Horizontal Comparison of Concordance and Robustness of LLMs

To further evaluate the potential performance differences between DeepSeek model and leading models Gemini 2.0 Pro and ChatGPT-4o in following expert consensus, this study conducted a comprehensive comparative analysis from multiple dimensions including consistency and robustness.

The concordance analysis showed that there was no significant difference in the overall concordance rate among the three tested models (*p* = 0.849), and the concordance rate of all models remained between 60% and 64%. Subgroup analysis of binary and nonbinary questions showed no significant difference in concordance rates, absolute proportional differences, or Pearson correlation coefficient *r* among the models (all *p* > 0.05). These results indicate that DeepSeek has comparable concordance with other evaluated models (Table 3).

**Table 3:** Horizontal comparison of consistency among large language models

	DeepSeek-V3	ChatGPT-4o	Gemini 2.0 Pro	*p*-Value
Overall concordance rate (N = 139)	88 (63.31%)	84 (60.43%)	88 (63.31%)	0.849^1^
*Binary questions (N = 37)*				
Common responses	25 (67.56%)	29 (78.38%)	23 (62.16%)	0.305^1^
Absolute proportional difference^3^	0.30 (0.13, 0.50)	0.24 (0.12, 0.34)	0.22 (0.13, 0.37)	0.397^2^
*Nonbinary questions (N = 102)*				
Common responses	63 (61.76%)	55 (53.92%)	65 (63.73%)	0.319^1^
Pearson correlation coefficient *r*^3^	0.82 (0.20, 0.98)	0.65 (−0.05, 0.94)	0.72 (0.25, 0.96)	0.117^2^

^1^Pearson’s chi-squared test

^2^Kruskal–Wallis test

^3^Median (M), interquartile range (P25, P75)

**Fig. 1:**
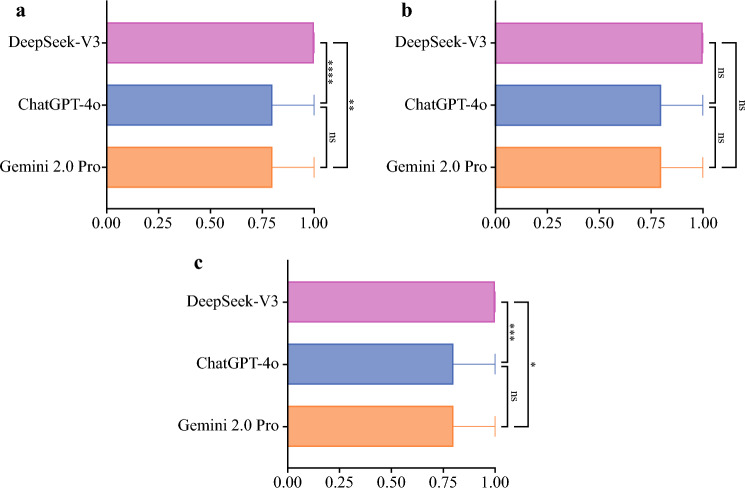
Comparison of robustness among large language models; **a** overall robustness across all consensus questions, **b** robustness for binary questions, **c** robustness for non-binary questions;* x*-axis represents the large language models tested, including DeepSeek-V3, ChatGPT-4o, and Gemini 2.0 Pro, and* y*-axis represents the robustness of the large language models, defined as the percentage of times the model selected its most frequent answer in 5 (or 6) runs; statistical comparisons were performed by the Kruskal–Wallis test, and post hoc pairwise comparisons were assessed by the Dwass–Steel–Critchlow–Fligner method; *p < 0.05; **p < 0.01; ***p < 0.001; ****p < 0.0001; *ns* not significant

The robustness analysis showed that all tested LLMs exhibited median robustness values ranging from 0.80 to 1.00, while the median voting rate of the expert panel on the top-voted answers was 0.60. The DeepSeek-V3 achieved a median robustness of 1.00 (IQR 0.80, 1.00), which is significantly better than Gemini 2.0 Pro (*p* = 0.005) and ChatGPT-4o (*p* < 0.001). There is no significant difference between Gemini 2.0 Pro and ChatGPT-4o (*p* = 0.557). In addition, subgroup analysis of multiclassification problems also revealed a similar trend, and DeepSeek models maintained their robustness advantage (Table 4, Fig. 1).

**Table 4:** Horizontal comparison of robustness among large language models

	DeepSeek-V3^1^	ChatGPT-4o^1^	Gemini 2.0 Pro^1^	*p*-Value^2^
Overall performance (N = 139) ^***3***^	1.00 (0.80, 1.00)	0.80 (0.60, 1.00)	0.80 (0.60, 1.00)	< 0.001*
*Post hoc pairwise comparisons p-values *^***4***^				
Versus DeepSeek-V3	–	< 0.001*	0.005*	
Versus ChatGPT-4o	–	–	0.557	
Versus Gemini 2.0 Pro	–	–	–	
Binary questions (N = 37) ^***3***^	1.00 (0.80, 1.00)	0.80 (0.60, 1.00)	0.80 (0.60, 1.00)	0.083
*Post hoc pairwise comparisons p-values *^***4***^				
Versus DeepSeek-V3	–	0.133	0.114	
Versus ChatGPT-4o	–	–	0.993	
Versus Gemini 2.0 Pro	–	–	–	
Nonbinary questions (N = 102)^3^	1.00 (0.60, 1.00)	0.80 (0.60, 1.00)	0.80 (0.60, 1.00)	< 0.001*
Post hoc pairwise comparisons *p*-values^4^				
Versus DeepSeek-V3	–	< 0.001*	0.036*	
Versus ChatGPT-4o	–	–	0.431	
Versus Gemini 2.0 Pro	–	–	–	

^1^Represents the robustness of the large language models, defined as the percentage of times the model selected its most frequent answer in five (or six) runs

^2^Kruskal–Wallis test

^3^Median (M) and interquartile range (P25, P75) of robustness

^4^Dwass–Steel–Critchlow–Fligner test

^*^Statistical significance was indicated as *p* < 0.05

### Horizontal Comparison by Topic Classification

**Fig. 2:**
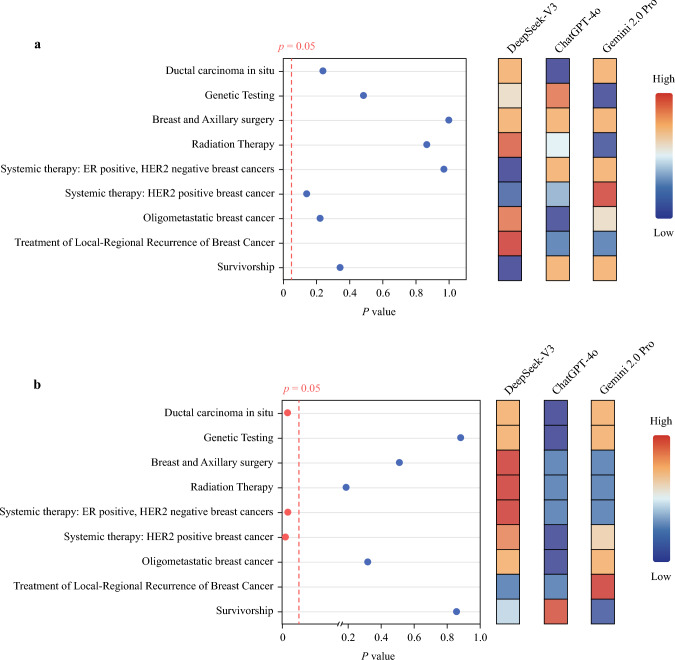
Comparison of large language models performance by clinical topic classification; **a** comparison of concordance among Large Language Models by clinical topic classification, **b** comparison of robustness among Large Language Models by clinical topic classification; *x*-axis represents the *p*-value of the comparison between models after the Pearson’s chi-squared test (in panel A) and after the Kruskal–Wallis test (in panel B) and *y*-axis represents the different clinical topics; each cell in the heatmap reflects either the concordance rate (panel A) or robustness score (panel B) for different clinical topics; color intensity corresponds to the magnitude of the value; statistical comparisons were performed by Pearson’s chi-squared test (in panel A) and Kruskal–Wallis test (in panel B); the number of questions on the subject “Treatment of locally recurrent breast cancer” is too small, thus no statistical analysis is required; *x*-axis has been expanded in the 0–0.2 range to emphasize differences in *p*-values in panel B; an axis break is indicated

Furthermore, we performed a stratified analysis of the concordance rate and robustness of each model in nine clinical topics. The concordance analysis showed that there was no significant difference in the consistency rate between models in different topics (all *p* > 0.05) (Fig. 2).

However, we observed heterogeneity in model robustness. In the topic of “Ductal carcinoma in situ,” DeepSeek-V3 showed superior robustness with a median of 1.00 (IQR 0.95, 1.00), significantly better than ChatGPT-4o (*p* = 0.048). In the topics of “Systemic therapy: ER positive, HER2 negative breast cancers” and “Systemic therapy: HER2 positive breast cancer,” DeepSeek-V3 showed better robustness than ChatGPT-4o (*p* = 0.016 and *p* = 0.003, respectively). The number of questions on the topic of “Treatment of local-regional recurrence of breast cancer” was too small, so only the median was shown and no statistical analysis was required. It is noteworthy that no single model performs best on all topics, suggesting that there may be differences in the performance of LLMs on different medical topics, which may be due to differences in the distribution of training data and the complexity of the problems^31^ (Table 5).

**Table 5:** Comparison of robustness among large language models by subject classification

	DeepSeek-V3^1^	ChatGPT-4o^1^	Gemini 2.0 Pro^1^	*p*-Value^2^
Topic “Ductal carcinoma in situ” (N** = 10)**^3^	1.00 (0.95, 1.00)	0.80 (0.60, 1.00)	1.00 (1.00, 1.00)	0.012*
Post hoc pairwise comparisons *p*-values^**4**^				
Versus DeepSeek-V3	–	0.048*	0.878	
Versus ChatGPT-4o	–	–	0.042*	
Versus Gemini 2.0 Pro	–	–	–	
Topic “Genetic testing” (N = 17)^3^	1.00 (0.80, 1.00)	0.80 (0.80, 1.00)	1.00 (0.60, 1.00)	0.882
Post hoc pairwise comparisons *p*-values^4^				
Versus DeepSeek-V3	–	0.993	0.892	
Versus ChatGPT-4o	–	–	0.918	
Versus Gemini 2.0 Pro	–	–	–	
Topic “Breast and axillary surgery” (N = 16)^3^	0.90 (0.65, 1.00)	0.80 (0.60, 1.00)	0.80 (0.65, 0.95)	0.511
*Post hoc pairwise comparisons p-values*^*4*^				
Versus DeepSeek-V3	–	0.566	0.603	
Versus ChatGPT-4o	–	–	0.953	
Versus Gemini 2.0 Pro	–	–	–	
Topic “Radiation therapy” (N = 28)^3^	0.90 (0.80, 1.00)	0.80 (0.60, 1.00)	0.80 (0.60, 1.00)	0.194
*Post hoc pairwise comparisons p-values*^*4*^				
Versus DeepSeek-V3	–	0.526	0.183	
Versus ChatGPT-4o	–	–	0.706	
Versus Gemini 2.0 Pro	–	–	–	
Topic “Systemic therapy: ER positive, HER2 negative breast cancers” (N = 44)^3^	1.00 (0.60, 1.00)	0.60 (0.60, 1.00)	0.60 (0.60, 1.00)	0.013*
*Post hoc pairwise comparisons p-values*^*4*^				
Versus DeepSeek-V3	–	0.016*	0.074	
Versus ChatGPT-4o	–	–	0.730	
Versus Gemini 2.0 Pro	–	–	–	
Topic “Systemic therapy: HER2 positive breast cancer” (N = 10)^3^	1.00 (0.80, 1.00)	0.60 (0.60, 0.80)	0.90 (0.60, 1.00)	0.006*
*Post hoc pairwise comparisons p-values*^*4*^				
Versus DeepSeek-V3	–	0.003*	0.474	
Versus ChatGPT-4o	–	–	0.142	
Versus Gemini 2.0 Pro	–	–	–	
Topic “Oligometastatic breast cancer” (N = 7)^3^	1.00 (0.80,1.00)	0.80 (0.80,1.00)	1.00 (0.80,1.00)	0.319
*Post hoc pairwise comparisons p-values*^*4*^				
Versus DeepSeek-V3	–	0.417	> 0.999	
Versus ChatGPT-4o	–	–	0.417	
Versus Gemini 2.0 Pro	–	–	–	
Topic “Treatment of local-regional recurrence of breast cancer” (N = 2)^5^	0.60	0.60	0.90	–
Topic “Survivorship” (N = 5)^3^	1.00 (0.70,1.00)	1.00 (0.70,1.00)	0.50 (0.50,1.00)	0.857
*Post hoc pairwise comparisons p-values*^*4*^				
Versus DeepSeek-V3	–	> 0.999	0.884	
Versus ChatGPT-4o	–	–	0.884	
Versus Gemini 2.0 Pro	–	–	–	

^1^Represents the robustness of the LLMs, defined as the percentage of times the model selected its most frequent answer in five (or six) runs

^2^Kruskal–Wallis test

^3^Median (M) and interquartile range (P25, P75) of robustness

^4^Dwass–Steel–Critchlow–Fligner test

^5^The number of questions related to the topic “Treatment of local-regional recurrence of breast cancer” was too small. Only the median is reported, and statistical analysis was not performed

^*^Statistical significance was indicated as *p* < 0.05

### Comparison of Concordance between LLMs and Expert Consensus across Evidence Levels

To further assess how evidence strength affects concordance between LLMs and expert consensus, we performed a sub-analysis on the basis of the concentration of expert voting. We divided the 139 questions into two groups: evidence-based questions and experience-based questions. Evidence-based questions were those with ≥ 80% expert voting rate and were generally addressed clearly in the guidelines (*n* = 25). Experience-based questions had < 80% expert voting rate and were not directly answered in the guidelines, requiring judgment on the basis of expert clinical experience (*n* = 114).

**Fig. 3:**
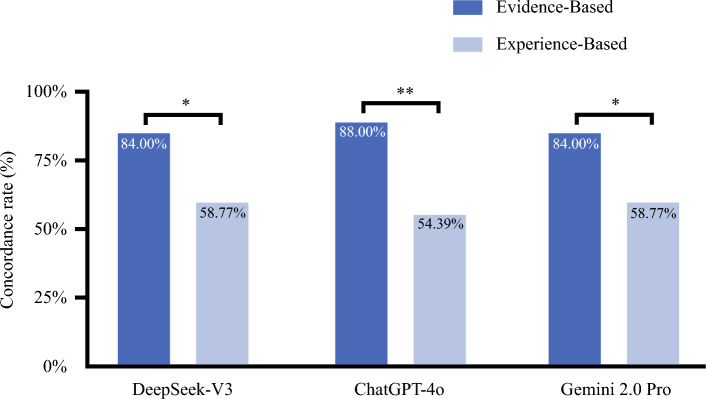
Comparison of concordance between LLMs and expert consensus across evidence levels; *x*-axis represents the large language models tested, including DeepSeek-V3, ChatGPT-4o, and Gemini 2.0 Pro and *y*-axis represents the concordance rate (%) of each LLM with the expert consensus; bars: each bar indicates the concordance rate for a specific model within a specific evidence category; dark blue bars represent the “Evidence-based” questions (i.e., questions with ≥ 80% expert voting rate), and light blue bars represent the “Experience-based” questions (i.e., questions with < 80% expert voting rate); numerical labels: the percentage value for each concordance rate is displayed directly within the corresponding bar for clarity; statistical analysis: statistical comparisons between the “Evidence-based” and “Experience-based” groups within each model were performed using the Pearson’s chi-squared test; the results of these comparisons are indicated by the significance markers (asterisks) above the bars; *p < 0.05, **p < 0.01

As shown in Fig. 3, all three models achieved higher concordance rate on evidence-based questions than on experience-based questions. DeepSeek-V3 reached 84.00% concordance rate for evidence-based questions and 58.77% for experience-based questions (*p* = 0.018). ChatGPT-4o showed a similar pattern (88.00% versus 54.39%, *p* = 0.002), as did Gemini 2.0 Pro (84.00% versus 58.77%, *p* = 0.018). These results suggest that LLMs perform well in areas supported by strong evidence but perform less concordance when clinical decisions depend mainly on expert experience.

## Discussion

This study systematically evaluated the emerging LLM DeepSeek model in terms of concordance with the expert panel consensus and the robustness of its response in clinical decision recommendations, using the discussion questions of the 19th SG-BCC meeting as a benchmark. We compared its performance with internationally representative models Gemini 2.0 Pro and ChatGPT-4o.

The results showed that DeepSeek-V3 had a concordance rate of 63.31% with the experts panel consensus. This performance was not significantly different from Gemini 2.0 Pro and ChatGPT-4o, indicating that DeepSeek exhibited a comparable capacity to interpret and agree with complex medical consensus. In addition, the overall concordance rate of all tested models (60–64%) was slightly improved compared with previous similar studies,^30^ which may be attributed to the developers’ continuous optimization work. However, the current concordance level is still far from the needs of clinical applications, and the models still face challenges in simulating expert decision-making on complex clinical problems.

In terms of robustness, the median response robustness of all tested models reached or exceeded 80% (0.8). Among them, DeepSeek-V3 performed particularly well, with a significantly higher robustness than other models. This suggests that the model can maintain consistent output when facing repeated queries. Although high robustness is of great benefit to tasks that require repetition, such as batch generation of standardized documents, in the field of clinical decision support, it does not mean high accuracy,^32^ and the model may stably give answers that do not conform to best clinical practice.^32–34^ Therefore, the high robustness of DeepSeek only reflects the stability of its output and does not directly demonstrate its clinical decision-making abilities.^35^

This study showed that the median voting rate of the expert panel on the top-voted answers was 60% (0.60), highlighting the inherent diversity of clinical opinions. This highlights the uncertainty and diversity of perspectives in real-world practice. It is worth noting that the internal robustness exhibited by the LLMs exceeds the level of expert panel consensus, indicating that there is a potential risk in directly equating its output results with established expert opinions. To better interpret this finding, it is necessary to distinguish consensus-based decision-making from algorithm-driven guidelines. SG-BCC reflects the heterogeneity of expert opinions. In areas where there are not currently many clinical trials, multiple clinical management approaches may be acceptable. NCCN guidelines are presented in flowchart form. It is not a simple vote, but a decision-making process based on the results of numerous clinical trials. According to the results in Fig. 3, all models can answer evidence-based questions on the basis of algorithm-driven guidelines with a higher concordance rate than experience-based questions on the basis of consensus-based decision-making. The design mechanism of LLM may be the reason. LLMs are designed to utilize their own material library and user-uploaded data to find answers with the optimal probability of matching keywords in the user's questions.^36,37^ When addressing evidence-based questions, clear guidelines allow LLMs to identify and reproduce a high-probability answer. In contrast, experience-based questions contain diverse and sometimes conflicting expert views, making it difficult for LLMs to determine a consistent response.

This difference reflects a significant limitation of LLMs. While they can effectively summarize and integrate structured guideline information, they currently fail to reflect the nuances of clinical judgment. These differences often arise in areas with limited or conflicting evidence, and they help surgeons develop personalized treatment plans for specific patient groups. Therefore, at this stage, LLMs should be strictly limited to auxiliary roles,^38–40^ such as retrieving disease knowledge, displaying guideline information, or generating preliminary plans. They should not completely replace clinicians’ professional judgment tailored to individual patient needs.^41,42^

In addition, this study found topic differences in the application performance of the models. The performance of the models may be affected by the coverage of specific domain knowledge in their training corpus. This highlights the need to subdivide the domain and design scenario-specific tests when evaluating and deploying such models in the medical field.^31^

This study has several limitations. First, the research questions were derived from a single international conference consensus, which inevitably led to limited coverage and restricted the testing scenarios of the models. Second, the evaluation method was confined to text-based question answering, while real-world clinical practice requires integration of multimodal data (such as imaging results, ultrasound results, and pathology results). This may affect the effectiveness of the model in real-world applications. Third, our research methodology provides the model with a limited set of reference guidelines, which aims to ensure the standardization of the testing environment and reduce errors caused by the model accessing different guidelines with varying priorities.^43^ At the same time, surgeons and patients often lack the time and resources to download a large number of guidelines and input them into LLM, so our study simulates real clinical scenarios to some extent. However, future research should undoubtedly explore the impact of providing a wider range of clinical guidelines on model performance. Furthermore, although the study evaluated concordance and robustness, it did not directly evaluate the clinical plausibility or safety of the model output.^44^

Future research in this field should monitor the progress of LLMs and establish a comprehensive and stable evaluation framework through continuous assessment. This system should rigorously review the clinical efficacy, security, and privacy safeguards of the model.^41,45^ At the same time, prospective research should be conducted under a strict ethical and regulatory framework to objectively evaluate the application value and potential risks of such models in actual medical scenarios.^46–48^

## Conclusions

The results of this study show that DeepSeek exhibited moderate concordance in following the 19th SG-BCC expert panel consensus, and its performance is comparable to leading international models such as Gemini 2.0 Pro and ChatGPT-4o. Furthermore, DeepSeek significantly outperformed other models in terms of answer robustness, highlighting its potential for application in clinical decision-making for breast cancer. In addition, given that all LLMs output results have moderate concordance but higher robustness than the expert panel, it is necessary to rigorously validate the clinical decision-making results output by LLMs.

## Supplementary Information

Below is the link to the electronic supplementary material.

Supplementary file1 (DOCX 19 KB)

Supplementary file2 (DOCX 14 KB)
