# Supplementary material for: St. Gallen International Breast Cancer Consensus-Based Clinical Decision Validation: Concordance Assessment Between Deep Large Language Model Outputs and Global Expert Panel Recommendations
Source: Ann Surg Oncol. 2026 Feb 10;33(5):4518–29. doi: 10.1245/s10434-026-19176-1 (PMC13083474; doi:10.1245/s10434-026-19176-1)
Supplement: Supplementary file 1 — Supplementary file1 (DOCX 19 KB) [file 10434_2026_19176_MOESM1_ESM.docx]

**Supplementary Table 1 Detailed Comparison of DeepSeek-R1 with the 19th St. Gallen International Breast Cancer Conference Expert Panel**

|  | **DeepSeek-R1** | **Expert Panel** |
| --- | --- | --- |
| **Overall Performance (N=139)** | | |
| Common responses | 85 (61.15%) | |
| Average robustness / majority in all questions (N=139) | 85.40% | 62.01% |
| Average robustness / majority in common answers (N=85) | 89.88% | 65.62% |
| Questions answered with average robustness / majority of≥80% | 102 (73.38%) | 25 (17.99%) |
| **Performance by question type** | | |
| **Binary questions (N=37)** | | |
| Common responses | 20 (54.05%) | |
| absolute proportional difference ***^1^*** | 0.40 (0.24, 0.57) | |
| **Non-Binary questions (N=102)** | | |
| Common responses | 65 (63.73%) | |
| Pearson correlation coefficient r ***^1^*** | 0.77 (0.24, 0.96) | |
| **Comparison of robustness among LLMs by topic classification** | | |
| Topic “Systemic therapy: ER positive, HER2 negative breast cancers” (N=44) | | |
| Common responses | 20 (45.45%) | |
| Average robustness / majority in this topic | 82.05% | 58.34% |
| Topic “Genetic testing” (N=17) | | |
| Common responses | 13 (76.47%) | |
| Average robustness / majority in this topic | 78.82% | 67.71% |
| Topic “Radiation therapy” (N=28) | | |
| Common responses | 16 (57.14%) | |
| Average robustness / majority in this topic | 88.21% | 64.04% |

***^1^***: Median (M) and interquartile range (P25, P75) of the above indicators.

Abbreviation. LLMs: large language models

Note: in this table, most of the measurement data are not normally distributed, but robustness is reported in the form of mean to reflect the specific differences without statistical inference. Absolute proportional difference and Pearson correlation coefficient r are still expressed in median and interquartile range.
