# Supplementary material for: St. Gallen International Breast Cancer Consensus-Based Clinical Decision Validation: Concordance Assessment Between Deep Large Language Model Outputs and Global Expert Panel Recommendations
Source: Ann Surg Oncol. 2026 Feb 10;33(5):4518–29. doi: 10.1245/s10434-026-19176-1 (PMC13083474; doi:10.1245/s10434-026-19176-1)
Supplement: Supplementary file 2 — Supplementary file2 (DOCX 14 KB) [file 10434_2026_19176_MOESM2_ESM.docx]

**Detailed Explanation of the Large-Scale Language Models Evaluated**

ChatGPT-4o is developed by OpenAI and a widely recognized multimodal model. It has been extensively evaluated across numerous medical specialties. For instance, they have demonstrated high accuracy in answering questions from medical licensing examinations^1^, showed promise in drafting radiology reports^2^, and have been assessed for their utility in ophthalmology and emergency medicine decision support^3,4^.

Gemini 2.0 Pro is a flagship model from Google and has also been a subject of intense academic scrutiny. Its advanced multimodal and reasoning capabilities have been benchmarked against expert clinicians in complex diagnostic challenges. Published research has shown its potential in interpreting medical imaging and providing differential diagnoses, often performing at a level comparable to human experts in specific, well-defined tasks^5,6^.

The DeepSeek model is a newly developed large language model from DeepSeek AI with lower usage and development costs and has also performed well in other medical fields. According to the developer's documentation, two models are provided, and each has its own characteristics. DeepSeek-V3 is a general-purpose model optimized for fast and reliable performance on common tasks such as information retrieval and summarization, while DeepSeek-R1 is designed for tasks requiring complex reasoning^7^. To date, the relative capabilities of the two models in clinical decision-making for breast cancer have not been determined, so we have included both in the study for evaluation, and the better-performing model will be compared with Gemini 2.0 Pro and ChatGPT-4o.

***References***

1. Gilson A, Safranek CW, Huang T, et al. How Does ChatGPT Perform on the United States Medical Licensing Examination (USMLE)? The Implications of Large Language Models for Medical Education and Knowledge Assessment. *JMIR Med Educ*. 2023;9:e45312. doi:10.2196/45312

2. Parillo M, Vaccarino F, Beomonte Zobel B, Mallio CA. ChatGPT and radiology report: potential applications and limitations. *Radiol Med*. 2024;129(12):1849-1863. doi:10.1007/s11547-024-01915-7

3. Waisberg E, Ong J, Kamran SA, et al. Generative artificial intelligence in ophthalmology. *Surv Ophthalmol*. 2025;70(1):1-11. doi:10.1016/j.survophthal.2024.04.009

4. Hoppe JM, Auer MK, Strüven A, Massberg S, Stremmel C. ChatGPT With GPT-4 Outperforms Emergency Department Physicians in Diagnostic Accuracy: Retrospective Analysis. *J Med Internet Res*. 2024;26:e56110. doi:10.2196/56110

5. Sozer A, Sahin MC, Sozer B, et al. Do LLMs Have “the Eye” for MRI? Evaluating GPT-4o, Grok, and Gemini on Brain MRI Performance: First Evaluation of Grok in Medical Imaging and a Comparative Analysis. *Diagnostics (Basel)*. 2025;15(11):1320. doi:10.3390/diagnostics15111320

6. Xu P, Wu Y, Jin K, Chen X, He M, Shi D. DeepSeek-R1 outperforms Gemini 2.0 Pro, OpenAI o1, and o3-mini in bilingual complex ophthalmology reasoning. *Adv Ophthalmol Pract Res*. 2025;5(3):189-195. doi:10.1016/j.aopr.2025.05.001

7. Sandmann S, Hegselmann S, Fujarski M, et al. Benchmark evaluation of DeepSeek large language models in clinical decision-making. *Nat Med*. 2025;31(8):2546-2549. doi:10.1038/s41591-025-03727-2
